# Discovery of rhynchophylline and mitraphylline in two Thai Mitragyna species and the investigation of their biological activity via opioid gene expression analysis

**DOI:** 10.1038/s41598-025-89715-5

**Published:** 2025-02-18

**Authors:** Runglawan Sudmoon, Tawatchai Tanee, Warin Wonok, Unchaleeporn Ameamsri, Thomas Liehr, Sakda Daduang, Pornnarong Siripiyasing, Arunrat Chaveerach

**Affiliations:** 1 Faculty of Law, Khon Kaen University, Khon Kaen, Thailand; 2 Faculty of Environment and Resource Studies, Mahasarakham University, Maha Sarakham, Thailand; 3 Department of Biology, Faculty of Science, Khon Kaen University, Khon Kaen, Thailand; 4 Institute of Human Genetics, Jena University Hospital, Jena, Germany; 5 Division of Pharmacognosy and Toxicology, Faculty of Pharmaceutical Sciences, Khon Kaen University, Khon Kaen, Thailand; 6 Faculty of Science and Technology, Rajabhat Mahasarakham University, Maha Sarakham, Thailand

**Keywords:** Rhynchophylline, Mitraphylline, Opioid receptor gene, *Mitragyna* species, Biochemistry, Chemical biology

## Abstract

*Mitragyna speciosa* (*Ms*), *M. diversifolia* (*Md*), *M. hirsuta* (*Mh*) and *M. rotundifolia* (*Mr*) were investigated for phytochemicals by GC-MS and GC-FID, cytotoxicity and genotoxicity testing by MTT and comet assay, and biological activity examination through gene expression of human µ, δ, κ, and nociceptin opioid receptors by qRT-PCR. The opioid substances mitragynine, 7-hydroxymitragynine, and mitraphylline were found in all studied species, and, for first time, rhynchophylline was found in *Mr*, and mitraphylline in *Md*, *Mh* and *Mr*. The MTT and comet assays of the ethanol and hexane leaf extracts on PBMCs revealed no cytotoxicity and no significant genotoxicity compared to the negative control, except for the hexane leaf extract of *Mh*,* which* caused significant DNA damage. The biological activity of the ethanolic extract of the four species showed a binding affinity to the µ (MOR) receptor revealing a relative gene expression of 89.54 and 50.41 by *Ms* and *Md* at 1.92 and 1.133 mg/ml, 32.42 and 19.97 mg/ml by *Md* and *Ms* at 3.77 and 1.76 mg/ml. *Mr* contained the three opioids mentioned plus rhynchophylline and showed low relative µ (MOR) gene expression of 16.89 at 0.189 mg/ml, while as an additional species, *Ipomoea aquatica* (*Ia*) showed higher relative µ (MOR) gene expression of 37.75 and 59.76 at 2.878, and 5.813 mg/ml. A combination of *Mr* and *Ia* at 1.227, 2.907 and 0.0123, 0.0291 mg/ml extract showed high relative µ (MOR) gene expression at 71.01 and 21.71. These *Mitragyna* species and the combination (formula details are patent registered), substances and their biological activities can be used for the innovative production of new medicines and further clinical investigation.

## Introduction

There are several publications on many aspects of kratom (*Mitragyna speciosa)*, including its phytochemistry, usage, toxicity, and popularity in the species’ native area of Southeast Asia. The major substances which are also the main active ingredients are alkaloids called mitragynine, 7-hydroxymitragynine, and mitraphylline. However, there are more substances found in the species such as ajmalicine, corynantheidine, paynantheine, speciogynine, rhynchophylline, etc^1–4^. Takayama^5^ reported that environmental factors play an important role in modifying the alkaloid content or other metabolites in the same species. So, the major substance mitragynine found in the leaves of *M. speciosa* in Thailand is 66.2% of all alkaloid content, higher than Malaysia’s which showed 12%. Additionally, young plants were found to have higher mitragynine levels than old trees. In Thailand and Malaysia, the leaves have been habitually consumed by natives and laborers where low doses (1–5 g) produced mild stimulation and euphoria for long-lasting work effects either by chewing the fresh leaves or making tea, and high doses (5–15 g) produced opioid-like effects. In traditional medicine, the plant has been used to treat diarrhea, cough, hypertension, relieve muscle pain and also commonly used as a substitute for opium^1,6^. Additionally, mitragynine from the *M. speciosa* leaves are used to stop opioid addiction^7,8^. In terms of the biological activity of alkaloids like mitragynine and its derivatives, they bind to opioid receptors leading *M. speciosa* to produce opioid-like effects such as sedation, euphoria, and analgesia resulting in the use of kratom leaves instead of drugs in the opioid group. Opioids are substances that act on opioid receptors to produce morphine-like effects. There are natural opiates such as morphine, codeine, and synthetic opioids such as fentanyl, carfentanil, protonitazene, and isotonitazene, methadone, meperidine, tramadol that all bind to opioid receptors^9^. Medically they are primarily used for pain relief, including anesthesia^10^. To date, five types of opioid receptors have been discovered– mu receptors (MOR), kappa receptors (KOR), delta receptors (DOR), nociception receptors (NOR) and zeta receptors (ZOR)^11^. Opioids have been divided into two types namely endogenous and exogenous opioids. Some endogenous opioids that bind to the receptors are enkephalins, endorphins, endomorphins, dynorphins, and nociception/orphanin. Exogenous opioids like morphine, heroin, and fentanyl are substances that are introduced into the body and bind to the same receptors as the endogenous opioids. The different types of opioid receptors that bind to their respective agonist counterparts showed different functions^11–13^ such as modulating antinociception and a variety of behavioral states such as anxiety, depression, and drug abuse expressed throughout the peripheral and central nervous systems^14^. The biological activity of *M. speciosa* leaves are caused by secondary metabolite alkaloids mainly mitragynine and 7-hydroxymitragynine which are additionally accountable for the pharmacological effects mentioned above^15^. The potency of these two substances in *M. speciosa* leaves are thought to be due to the agonist activity against mu- and kappa-opioid receptors^4^ and function as partial agonists of the human mu opioid receptor^3^.

Aside from *M. speciosa*, Thailand has more three *Mitragyna* species including *M. diversifolia*, *M. hirsuta* and *M. rotundifolia*^16^ which have very little research worldwide on their phytochemicals aside from the findings of triterpenoid saponins in *M. rotundifolia* from China^17^, the new trans- and cis-p-coumaroyl flavonol tetraglycosides in *M. rotundifolia* leaves from Thailand^18^, and the new heteroyohimbine-type oxindole alkaloid in *M. hirsuta* leaves from Thailand^19^. In addition to opioids, cannabinoids such as oleamide contained in *Ipomoea aquatica* are interesting for their ability to relieve stress, improve memory, induce deep sleep, improve appetite and be anti-inflammatory^20–23^. Therefore, the combination of *I. aquatica* and *M. rotundifolia* can create a formula that has beneficial effects for the elderly.

From the above review, it can be seen that most studies have been done on *M. speciosa*. Other species that are close phylogenetically in the same genus have not been studied.

Therefore, *M. speciosa* was included in this study to compare with the other three species in terms of chemicals, toxicity testing, and biological activity examined via the quantitative reverse transcription-polymerase chain reaction (qRT-PCR) method using the opioid genes. Additionally, the formula made with the combination of *M. rotundifolia* and *I. aquatica* was tested following the methods mentioned.

## Materials and methods

### Plant materials

The four *Mitragyna* species leaves were collected in Thailand and identified by the proficient botanist, Prof. Dr. Arunrat Chaveerach. The leaves of *M. speciosa* were purchased from a farm (now listed as a legal herb in Thailand) in Khon Kaen province. The other three species, *M. rotundifolia*, *M. hirsuta*, *M. diversifolia* and *Ipomoea aquatica* were collected from private areas in early 2022. The experimentation on plants is in accordance with relevant guideline and legislation. The specimens were kept at the herbarium of Department of Biology, Faculty of Science, Khon Kaen University with collector numbers A. Chaveerach 1105 (*M. speciosa*), A. Chaveerach 1106 (*M. rotundifolia*), A. Chaveerach 1107 (*M. hirsuta*), A. Chaveerach 1108 (*M. diversifolia*) and A. Chaveerach 936 (*I. aquatica*). The leaves were washed and air-dried at room temperature or dried in the oven at 60 °C. Once dried, they were kept in a place with low humidity until they were used.

### Plant extract preparation

The finely powdered leaves of the four *Mitragyna* species were soaked separately with ethanol and hexane solvents at a ratio of 1 g: 5 ml, incubated for 72 h at room temperature. When the time was up, the extracts were filtered through filter paper (Whatman no 1). The filtrates were further used for phytochemical analysis, toxicity testing and opioid gene expression analysis. The filtrates or extracts were kept at -20 °C until used. Another solution with a ratio of 1 g: 2.5 ml was also similarly prepared for opioid gene expression analysis. The objectives for the extraction ratios are to have various concentrations for the highest amount of opioid gene expression.

### Phytochemical screening by gas chromatography–mass spectrometry (GC-MS)

For phytochemical screening of the studied plants, the extracts were analyzed using GC-MS, an Agilent Technologies 5977B Inert Plus MSD/GC8890 fused with a capillary column HP-5MS (30.0 m × 0.25 mm × 0.25 μm). The extracts were filtered through a Whatman No. 1 and nylon syringe filter at pore size 0.45 μm. The 1 µl of the aliquot was injected in the capillary column with a split mode. Helium gas was used as the carrier at a constant flow rate of 1 ml/min. The injection and mass-transfer line temperature were set at 150 °C for 3 min, then the temperature was increased by 20 °C/minute to 300 °C and left for 9.5 min. The temperatures of the injection and mass transfer ports were set at 250 and 300 °C. The mass detector was set to scan mode (40–400 m/z) and ion source to EI mode. The relative percentage of phytochemical constituents was calculated as a percentage by peak area normalization. Components were identified by comparing the obtained mass spectra with reference compounds in the W11N17main.L library.

### Mitragynine, 7-hydroxymitragynine, mitraphylline and rhynchophylline detection, and quantification by gas chromatography-flame ionization detection (GC-FID)

For phytochemical analysis of the studied plants, mitragynine, 7-hydroxymitragynine, mitraphylline and rhynchophylline were examined by GC using an Agilent Technologies 7890B GC system, equipped with flame ionization detector (FID). The chromatographic conditions were almost identical for the four substances, but with the following differences in the parameters of constant flow rate/min, initial/last temperature of the column °C/min, and total timing. Helium was used as the carrier gas, at a flow rate of 1.8, 1.8, 1.4, 1.5 ml/min, respectively. The injector and detector temperatures were 280 °C. The initial temperature for the column was set at 250 °C for 3, 2, 3 and 2 min and increased by 20 °C/min to a final temperature of 300 °C, which was held for 9.5, 6.5, 5.5 and 3 min, total time is 15, 12, 12, and 14 min. Then, 1 µL of each sample was injected into a HP-5 capillary column (30.0 m × 0.32 mm × 0.25 μm × 0.25 μm) with a split mode.

100 µg each of the standards, mitragynine and 7-hydroxymitragynine (Merck, Germany, HPLC grade), were dissolved with 1 ml methanol. The standard solutions were 2-fold serially diluted for 5 levels. They were filtered with a nylon syringe with pore size 0.45 μm. For each of the mitraphylline and rhynchophylline standards (Merck, Germany, HPLC grade), 1 mg was dissolved with 1 ml methanol. The standard solutions were 2-fold serially diluted for 5 levels. They were filtered with a nylon syringe with pore size 0.45 μm. These various standard concentrations and areas were applied to plot the calibration curve. The linear equation of y = mx + c and a correlation coefficient (R^2^) were calculated using Microsoft Excel. For quantification of the plant extracts mitragynine, 7-hydroxymitragynine, mitraphylline and rhynchophylline, the area of the extracts was substituted in y to calculate x as a concentration of the targeted substance.

### Cell preparation: normal human peripheral blood mononuclear cells (PBMC) isolation

Sodium heparin anticoagulated venous blood (buffy coat) samples were obtained from the blood bank of the Srinagarind Hospital, Faculty of Medicine, Khon Kaen University for PBMC isolation. PBMC preparation was performed following Sudmoon et al.^24^. The cells were used for toxicity testing and opioid gene expression analysis.

### Toxicity testing (cytotoxicity by (3-(4,5-dimethylthiazol-2-yl)-2,5-diphenyltetrazolium bromide assay, MTT assay and genotoxicity, comet assay)

Toxicity testing of the ethanol and hexane extracts of the four *Mitragyna* species at both the cell and genetic levels were performed using MTT and comet assays following Sudmoon et al.^24^.

### Cell treatment, RNA extraction and cDNA synthesis

To evaluate the effect on opioid gene receptor expression of the mu (µ), delta (δ), kappa (κ) and nociceptin receptors by mitragynine, 7-hydroxymytragynine, mitraphylline and rhynchophylline, the PBMCs (2 × 10^6^ cell/ml) were seeded into 24-well plates. The four plant extracts at the two ratios of extraction (1 g: 2.5 ml and 1 g: 5 ml) with the extract preparation and concentration are shown in Table 1.

**Table 1:** Ratio of extraction, extract concentration and working concentration for each of the studied species.

Sample name	Ratio of extraction (g plant: ethanol)	Extract concentration (mg/ml 100% DMSO)	Working concentration (mg/ml DMSO)
*Mitragyna speciosa*	1:5	17.60	1.76 (1×)
			0.176 (0.1×)
			0.0176 (0.01×)
	1:2.5	19.23	1.923 (1×)
			0.1923 (0.1×)
			0.01923 (0.01×)
*M*. *hirsuta*	1:5	15.60	1.560 (1×)
			0.1560 (0.1×)
			0.01560 (0.01×)
	1:2.5	6.50	0.650 (1×)
			0.0650 (0.1×)
			0.00650 (0.01×)
*M*. *diversifolia*	1:5	37.70	3.770 (1×)
			0.3770 (0.1×)
			0.03770 (0.01×)
	1:2.5	11.33	1.133 (1×)
			0.1133 (0.1×)
			0.01133 (0.01×)
*M*. *rotundifolia*	1:5	18.90	1.890 (1×)
			0.1890 (0.1×)
			0.01890 (0.01×)
	1:2.5	24.54	2.454 (1×)
			0.2454 (0.1×)
			0.02454 (0.01×)
*Ipomoea aquatica*	1:5	28.78	2.878 (1×)
			0.2878 (0.1×)
			0.02878 (0.01×)
	1:2.5	58.13	5.813 (1×)
			0.5813 (0.1×)
			0.05813 (0.01×)

The PBMCs were treated with all the three working concentrations mentioned above at x, 0.1x and 0.01x for 24 h at 37 °C. The untreated cell was used as a negative control. All experiments were performed in triplicate. After cell harvesting, the RNA extraction was performed according to manufacturer’s protocol using GF-1 Total RNA Extraction Kit (Vivantis, Malaysia). The concentration of total RNA was quantified with Nano spectrophotometer (DeNovix, USA). Subsequently, cDNA synthesis was performed using Kit Viva 2-step RT-PCR Kit with M-MuLV RT/Taq DNA Polymerase (Vivantis, Malaysia) using Oligo(dT)_18_ primer according to manufacturer’s protocol. The cDNA was kept at -20 °C until use.

### Gene expression analysis by quantitative real time polymerase chain reaction (qRT-PCR) in the four studied *Mitragyna* species

The qRT-PCR assay was performed using SYBR Green Master Mix (4x CAPITAL™ qPCR Green Master Mix, Biotechrabbit Co., Germany). Amplifications were carried out with the final reaction solution of 20 µL containing 5 µL SYBR Green Master Mix, a pair of forward and reverse primers (10 µM) each 0.5 µL, nuclease-free water 12 µL, and 2 µL of first-stranded cDNA template (5x diluted with nuclease-free water). PCR (LightCycle-^®^ 480 Instrument II Real-Time PCR System, Roche F. Hoffmann-La Roche AG Konzern-Hauptsitz Grenzacherstrasse 124 CH-4070 Basel Schweiz) conditions were programed following pre-denaturation at 95 °C for 10 min followed by 45 cycles of 95 °C for 15 s, 60 °C for 30 s, and 70 °C for 30 s, for denaturation, annealing and extension, respectively. The melting curve analysis was set to verify the specificity of each pair of primers, holding at 95 °C for 30 s, 60 °C for 2 min and a continuous rise in temperature to 95 °C at 0.11 °C/s ramp rate, and cooling at 40 °C for 30 s. For Mor, µ opioid receptor, 45 cycles comprised of 95 °C for 15 s, 58 °C for 60 s, and 72 °C for 60 s, for denaturation, annealing and extension, respectively. The melting curve analysis was set to verify the specificity of each pair of primers, following 95 °C for 30 s, 64 °C for 2 min and a continuous raise in temperature to 95 °C at 0.11 °C/s ramp rate, and cooling at 40 °C for 30 s. The cross-point cycle of threshold (Ct) in each treatment was applied to calculate using the formula 2^−ΔΔCP^^25^ for relative gene expression calculation. The expression of target genes was normalized by the GAPDH expression value. The sequences of the primers are listed in Table 2.

**Table 2:** The human opioid receptor primers (HORP) including forward and reverse sequences.

HORP	Forward (5ʹ-3ʹ)	Reverse (5ʹ-3ʹ)	Reference
GAPDH	GTCTCCTCTGACTTCAACAGCG	ACCACCCTGTTGTGTAGCCAA	Sudmoon et al.^23^
MOR, µ	TACCGTGTGCTATGGACTGAT	ATGATGACGTAAATGTGAATG	Escelsior et al.^35^
OPRM1, µ	CTGGTTGGAACATGAGAGCA	TGGCAGTCTTCATCTTGGTG	NM 001145287.3
OPRD1, δ	CCACTCACTGCCATCTCCT	AGTCCTGATGCCCAGAGTG	NM 000911.4
Delta	CTCATCATCACCGTGTGCTA	AGACGATGACGAAGATGTGG	U07882.2
Kappa	ATGATCCTGCGTCTCAAGAG	CGGAAACACCGCTTGAAGTT	Gunji et al.^36^
OPRK1, κ	GATCCCTGTCCTCATCATCA	GCCTCCACCAGGATGAAT	NM 001282904.2
OPRL1, nociceptin	TTTCCTGGTCTTGACTGCTC	TTCTCCTTCCACACAGCTTC	NM 182647.4

### Gene expression analysis by quantitative real time polymerase chain reaction (qRT-PCR) with the combination of *M. Rotundifolia* and *Ipomoea aquatica*

After GC-FID was performed and showed rhynchophylline in *M. rotundifolia*, supplemented with its previously published biological activity of inducing sleep^26^, and oleamide in *I. aquatica*, 0.74–1.71 mg/ml and 1.10–7.52 mg/g^27^ which relieves stress, induces deep sleep, improves memory, and is anti-inflammatory^20–23^, a two-plant formulation and ethanol extraction (1:2.5 and 1:5, g plant/solvent) was created (the ratio is in the patent) to determine the mixture’s opioid affinity. Details of the extraction ratio and the concentration used in qRT-PCR assays are patent registered and shown in Table 1.

## Results

Several phytochemicals of the four *Mitragyna* species *M. speciosa*, *M. diversifolia*, *M. hirsuata*, and *M. rotundifolia* found in Thailand, investigated by GC-MS were revealed and are listed in Table 3, their chromatograms are not shown. The major constituents of opioid substances are 36.89% mitragynine in the ethanolic *M. speciosa* extract and 18.38% in the ethanol *M. diversifolia* extract, rhynchophylline at 34.75% in the ethanol *M. rotundifolia* extract, mitraphylline 10.94% in the ethanol *M. hirsuta* extract. The other substances were found in small amounts except squalene which was found in relatively high quantities in all the studied species, 34.45% in the *M. speciosa* hexane extract, 22.73% in the *M. diversifolia* hexane extract, 16.94% in the *M. hirsuta* hexane extract, and 8.05% in the *M. rotundifolia* hexane extract.

**Table 3:** Summary of phytochemical constituents of the four *Mitragyna* species sorted by their relative content percentages, analyzed by GC-MS on ethanol (E) and hexane (H) extracts.

Compound	Formula	Relative content (%)*							
		M. diversifolia		M. rotundifolia		M. hirsuta		M. speciosa	
		E	H	E	H	E	H	E	H
Mitragynine	C_23_H_30_N_2_O_4_	18.38	-	-	-	-	-	36.89	-
Rhynchophylline	C_22_H_28_N_2_O_4_	-	-	34.75	-	-	-	-	-
Squalene	C_30_H_50_O	10.28	22.73	2.17	8.05	8.90	16.94	12.14	34.45
Mitraphylline	C_21_H_24_N_2_O_4_	-	-	-	-	10.94	-	-	-
Octadecane	C_18_H_38_	-	-	-	11.93	-	24.82	-	9.44
2-Methyltricosane	C_24_H_50_	-	10.37	1.52	28.54	-	-	-	7.56
γ-sitosterol	C_29_H_50_O	-	-	-	-	-	-	6.42	-
Phytol	C_20_H_40_O	5.86	9.25	3.29	1.46	13.35	5.89	6.35	4.59
Dodecanoic Acid	C_12_H_24_O_2_	15.51	-	5.81	-	8.58	-	5.66	-
1-(4-Amino-3- butoxyphenyl)ethanone	C_12_H_17_NO_2_	9.69	16.77	5.00	-	9.11	12.64	-	12.83
Paynantheine	C_23_H_28_N_2_O_4_	-	-	-	-	-	-	4.75	-
α-Tocopherol acetate	C_31_H_52_O_3_	-	-	-	10.51	2.26	-	-	-
Octadecanal	C_18_H_36_O	1.84	-	-	-	2.65	-	0.61	-
Vitamin E	C_29_H_50_O_2_	1.68	-	3.12	-	-	4.30	2.87	7.64
Heptadecanal	C_17_H_34_O	-	-	-	-	-	9.97	-	-
5-((3-(4-methoxybenzylideneamino) phenyl) diazenyl)-quinolin-8-ol	C_23_H_18_N_4_O_2_	-	-	19.98	-	-	-	-	-
Palmitic acid	C_16_H_32_O_2_	3.25	-	2.16	-	4.07	-	2.53	-
D-Ribose	C_5_H_10_O_5_	5.76	0.48	0.98	-	0.53	-	1.70	-
Cycasin	C_8_H_16_N_2_O_7_	1.24	-	2.48	-	1.93	-		-
3-phenylphthalimidine	C_14_H_11_NO	2.16	16.49	0.67	11.06	4.52	11.17	2.66	6.24
Glyceryl palmitate	C_19_H_38_O_4_	-	-	-	-	-	-	1.51	-
2,5-Di-tert-butylhydroquinone	C_14_H_22_O_2_	-	-	4.98	-	-	-	-	-
2-Aminoethanethiolsulfuric acid	C_2_H_7_NO_3_S_2_	0.45	0.31	1.99	0.84	2.01	-	0.83	0.89
Hexadecatrienoic acid, methyl ester	C_17_H_28_O_2_	1.16	-	0.38	-	1.41	-	0.82	0.82
Hexopyranose	C_6_H_12_O_6_	1.77		2.63		1.91		3.20	-
Ethyl linolenate	C_20_H_34_O_2_	-	-	-	-	-	-	1.52	-
5-(Phenoxy)-methyl-2-amino-1,3,4-thiadiazoles	C_9_H_9_N_3_OS	2.16	1.17	1.29	1.61	2.53	4.22	2.25	0.61
6,7-Dimethoxy-2-methyl-3,4-dihydro[1-D]isoquinolinium ion	C_12_H_15_DNO_2_	-	4.83	-	3.23	0.19	3.13	-	3.38
Nerolidyl acetate	C_17_H_28_O_2_	0.35	-	0.46	-	0.43	0.44	0.77	-
Tris(tert-butyldimethylsilyloxy)arsane	C_18_H_45_AsO_3_Si_3_	6.70	9.72	2.65	2.18	7.05	5.06	1.75	3.67
2,4-Di-tert-butylphenol	C_14_H_22_O	-	-	-	0.58	-	0.37	-	-
Arsenous acid, tris(trimethylsilyl) ester	C_9_H_27_AsO_3_Si_3_	1.10	5.60	0.59	5.89	1.59	0.58	-	6.23
Tetrahydroionone	C_13_H_24_O	0.57	0.58	-	-	0.53	-	-	-
Methyl 8,9-octadecadienoate	C_19_H_34_O_2_	0.72	-	-	-	1.36	-	1.25	0.51
N, N-Disalicylidene-1,2-diaminopropane	C_17_H_18_N_2_O_2_	-	-	-	-	3.44	-	-	-
alpha-D-Glucopyranoside, methyl	C_7_H_14_O_6_	-	-	0.60	-	-	-	-	-
Ethyl palmitate	C_18_H_36_O_2_	0.82	-	-	-	3.60	-	1.11	0.72
Ethyl stearate	C_20_H_40_O_2_	-	-	-	-	0.71	-	-	-
Ononitol	C_7_H_14_O_6_	0.35	-	-	-	0.34	-	-	-
Octamethylcyclotetrasiloxane	C_8_H_24_O_4_Si_4_	-	-	-	-	0.37	-	-	-
Globulol	C_15_H_26_O	4.88	-	-	-	0.38	-	0.38	-
1,2-Dimethyl-3,5-bis(1-methylethenyl)cyclohexane	C_14_H_24_	0.78	-	-	-	2.30	-	0.65	-
10-Methoxy-nb-alpha-methylcorynantheol	C_21_H_29_N_2_O_2_	-	0.33	0.63	-	0.62	-	-	-
Verbenone	C_10_H_14_O	0.67	-	-	-	-	-	-	-
Myristyl glyceryl ether	C_17_H_36_O_3_	-	-	-	-	0.41	-	-	-
1-methyl-3-oxidanyl-3- (1-phenylpropa-1,2- dienyl)indol-2-one	C_18_H_15_NO_2_	-	-	-	-	-	-	0.53	-
2-Butoxy-4-methyl- [1,3,2]dioxaborinane	C_8_H_17_BO_3_	0.59	-	-	-	-	-	-	-
5-Nitrobenzotriazole	C_6_H_4_N_4_O_2_	-	-	-	-	0.31	-	-	-
2,3-Bornanediol	C_10_H_18_O_2_	0.50	-	0.53		1.21	-	0.78	-
farnesol	C_15_H_26_O	-	-	-	0.51	-	-	-	-
Hexasiloxane, 1,1,3,3,5,5,7,7,9,9,11,1 1-dodecamethyl	C_12_H_38_O_5_Si_6_	0.33	0.83	1.36	13.59	0.32	0.47	0.46	0.44
Isocaucalol	C_15_H_26_O_3_	0.28	0.55	-	-	0.17	-	-	-
2-(5-aminotetrazol-1-yl)-acetic acid	C_3_H_5_N_5_O_2_	0.19	-	-	-	-	-	-	-

*E for ethanol extracts, H for hexane extracts.

**Fig. 1:**
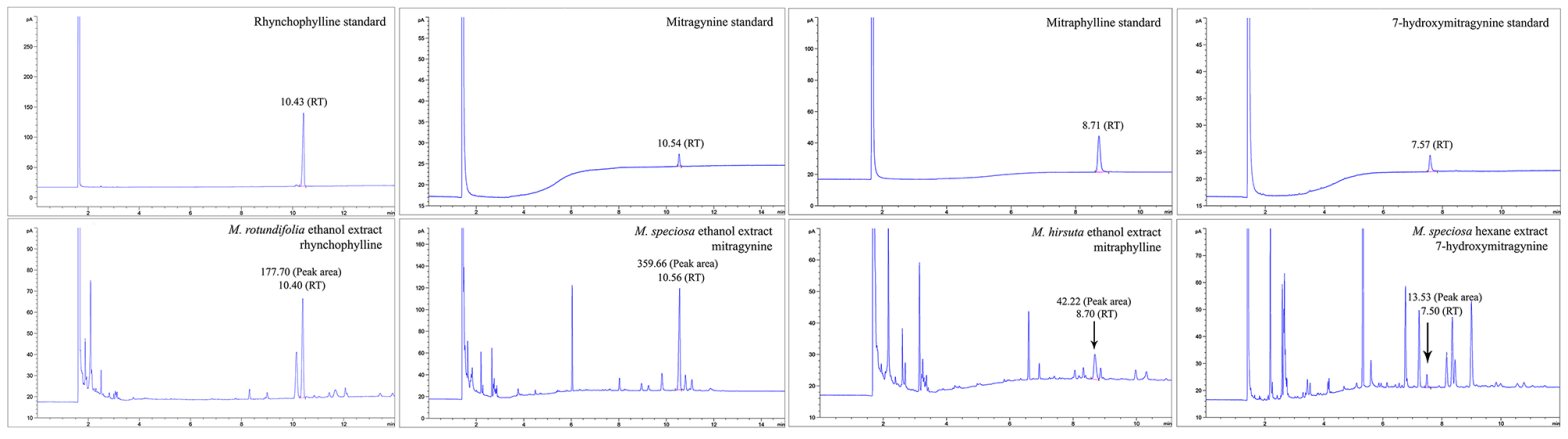
The representative GC-FID chromatograms showing peak areas and retention times of rhynchophylline, mitragynine, mitraphylline and 7-hydroxymitragynine standards, and the studied ethanol and hexane extracts with highest peak area of each investigated substance. Ethanol and hexane solvents were also subjected to this analysis for reference (chromatogram not shown).

When the research went through detailed measurement by GC-FID of mitragynine, 7-hydroxymitragynine, mitraphylline and rhynchophylline in both the ethanol and hexane extracts of the four studied *Mitragyna* species, mitragynine, 7-hydroxymitragynine, and mitraphylline were found in all the four species with varied quantities and concentrations, but rhynchophylline was found only in. *M. rotundifolia*. The GC-FID chromatogram of each substance (Fig. 1) revealed retention time and peak area according to each standard substance which produced a calibration curve with the linear equation y = 0.1058x + 0.1696, R^2^ = 0.998 for mitragynine; y = 0.1517x-0.235, R^2^ = 0.999 for 7-hydroxymitragynine; y = 132.66x – 0.6529, R^2^ = 0.999 for mitraphylline; and y = 274.82x + 5.5592, R^2^ = 0.999 for rhynchophylline. The area of the extracts was substituted in y to calculate x as a concentration and content of the targeted substances shown in Table 4.

**Table 4:** The summary of the quantities and concentrations of rhynchophylline, mitragynine, mitraphylline, and 7-hydroxymitragynine in the four studied *Mitragyna* species by GC-FID analysis.

Phytochemical	Mitragyna diversifolia		M. rotundifolia		M. hirsuta		M. speciosa	
	Ethanol	Hexane	Ethanol	Hexane	Ethanol	Hexane	Ethanol	Hexane
Rhynchophylline								
Peak area (pA*s)	ND	ND	177.7	ND	ND	ND	ND	ND
Conc. (mg/ml)	ND	ND	0.63	ND	ND	ND	ND	ND
Amount (mg/g plant)	ND	ND	0.94	ND	ND	ND	ND	ND
Mitragynine								
Peak area (pA*s)	1.74	1.12	1.08	1.38	2.03	1.94	359.66	2.92
Conc. (mg/ml)	0.01	0.01	0.01	0.01	0.02	0.02	3.4	0.03
Amount (mg/g plant)	0.03	0.03	0.01	0.03	0.04	0.05	5.27	0.07
Mitraphylline								
Peak area (pA*s)	19.01	4.31	26.45	5.12	42.22	4.07	28.15	6.42
Conc. (mg/ml)	0.14	0.03	0.19	0.03	0.31	0.03	0.21	0.04
Amount (mg/g plant)	0.27	0.08	0.29	0.1	0.66	0.07	0.32	0.11
7-Hydroxymitragynine								
Peak area (pA*s)	7.57	6.51	2.42	7.91	1.8	13.16	9.81	13.53
Conc. (mg/ml)	0.05	0.04	0.01	0.05	0.01	0.09	0.06	0.09
Amount (mg/g plant)	0.09	0.12	0.02	0.15	0.02	0.24	0.1	0.22

**Fig. 2:**
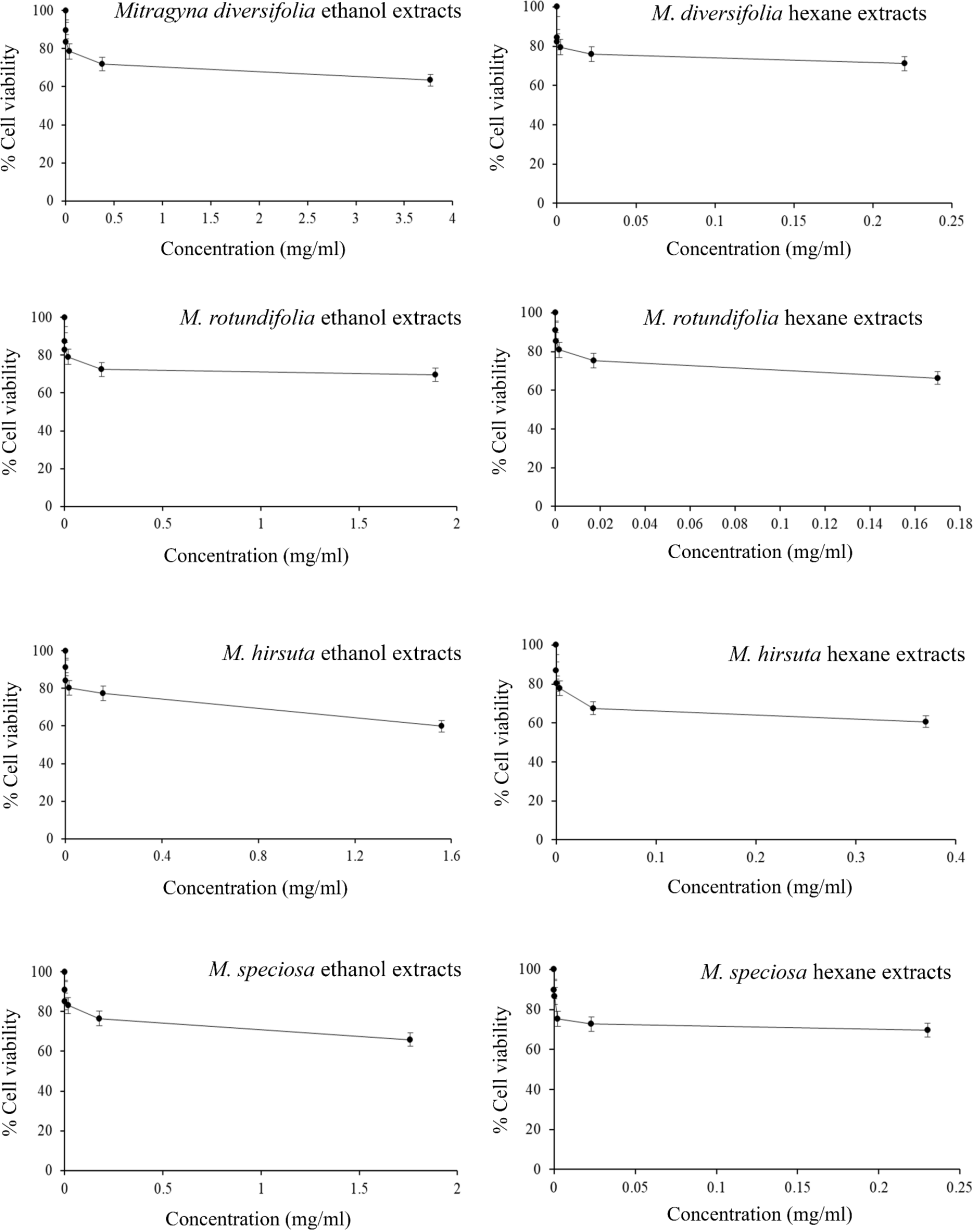
Graph based on the MTT assay of the studied plant concentrations showing cell viability percentage which did not have IC_50_ values meaning they did not have cytotoxicity on PBMCs treated with the *Mitragyna diversifolia*, *M. rotundifolia*, *M. hirsuta*, and *M. speciosa* ethanol and hexane extracts.

**Fig. 3:**
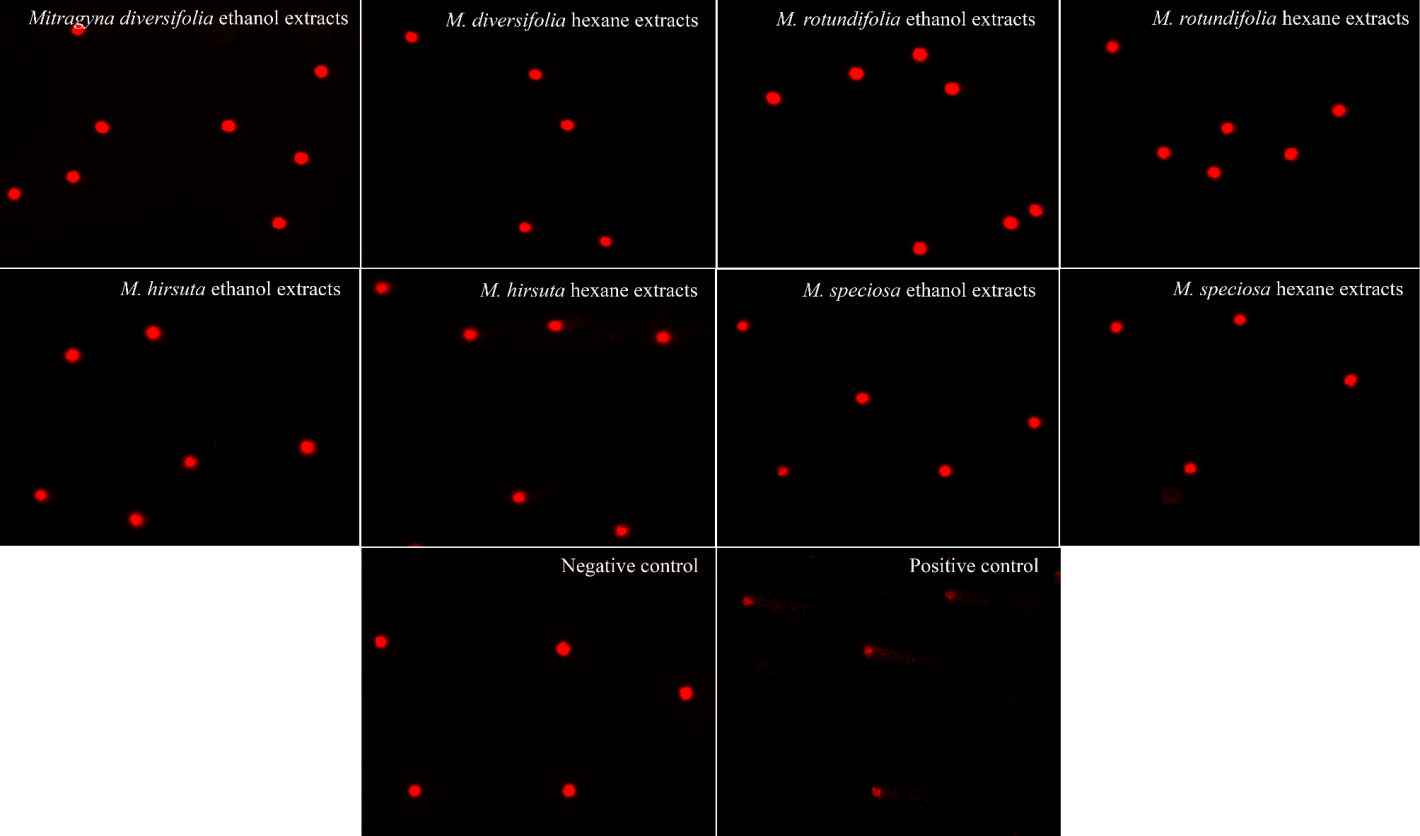
Result of genotoxicity evaluation with comet assay (200x) on PBMCs of the highest working concentration used in the MTT assay (when without IC_50_ value) of *Mitragyna diversifolia*, *M. rotundifolia*, *M. hirsuta*, and *M. speciosa* ethanol and hexane extracts. All four ethanol extracts and the hexane extracts of *M. diversifolia*, *M. rotundifolia*, and *M. speciosa* showed no significant DNA damage (*p* > 0.05), while the *M. hirsuta* hexane extract showed significant DNA damage (*p* < 0.05) compared to the negative control.

The results of the MTT assay did not have IC_50_ values with high percentages of cell viability shown on the graph of the extract concentrations (Fig. 2; Table 5). Following detailed toxicity testing on the DNA level via comet assay using the highest working concentrations when there was no IC_50_ value, the extract samples showed no significant DNA damage (*p* > 0.05) with the four ethanol plant extracts, and the three hexane extracts of *M. diversifolia*, *M. rotundifolia* and *M. speciosa* on PBMCs compared to the negative control. Only the *M. hirsuta* hexane extract showed significant DNA damage (*p* < 0.05) compared to the negative control (Figs. 2 and 3; Table 5).

**Table 5:** Summary of all details used in the MTT and comet assays on PBMCs, both for individual species and for the formula, showing high cell viability percentage without IC_50_ values in the MTT assay, and the level of DNA damage evaluated by comet assay based on Olive tail moment after treatment with the mentioned sample extracts showing no significant DNA damage (*p* > 0.05), and significant DNA damage (*p* < 0.05).

Plant	Solvent	Working concentration (mg/ml)	MTT essay	Comet essay	
			Cell viability (Percentage ± S.D.)	Olive tail moment (Median ± S.D.)	*p*-value
*Mitragyna diversifolia*	ethanol	3.77	63.43 ± 0.29–89.51 ± 0.52	0.54 ± 0.20	0.09
	hexane	0.22	71.09 ± 0.37–84.43 ± 0.52	0.53 ± 0.25	0.16
*M. rotundifolia*	ethanol	1.89	69.68 ± 0.32–87.57 ± 0.45	0.52 ± 0.23	0.39
	hexane	0.17	66.33 ± 0.20–91.17 ± 0.47	0.56 ± 0.23	0.10
*M. hirsuta*	ethanol	1.56	59.84 ± 0.31–91.44 ± 0.55	0.48 ± 0.21	0.45
	hexane	0.37	60.70 ± 0.24–86.99 ± 0.44	0.66 ± 0.24	< 0.05
*M. speciosa*	ethanol	1.76	65.95 ± 0.25–91.12 ± 0.40	0.47 ± 0.18	0.15
	hexane	0.23	69.64 ± 0.14–89.85 ± 0.38	0.50 ± 0.23	0.12
*M. speciosa*	ethanol	1.76	65.95 ± 0.25–91.12 ± 0.40	0.47 ± 0.18	0.15
	hexane	0.23	69.64 ± 0.14–89.85 ± 0.38	0.50 ± 0.23	0.12
*M*. *rotundifolia* + *I*. *aquatica* extracts	ethanol	1.227, 2.907	94.28 ± 0.18	1.6825 ± 0.6926	< 0.05
Control	-	-	-	0.45 ± 0.28	-

**Fig. 4:**
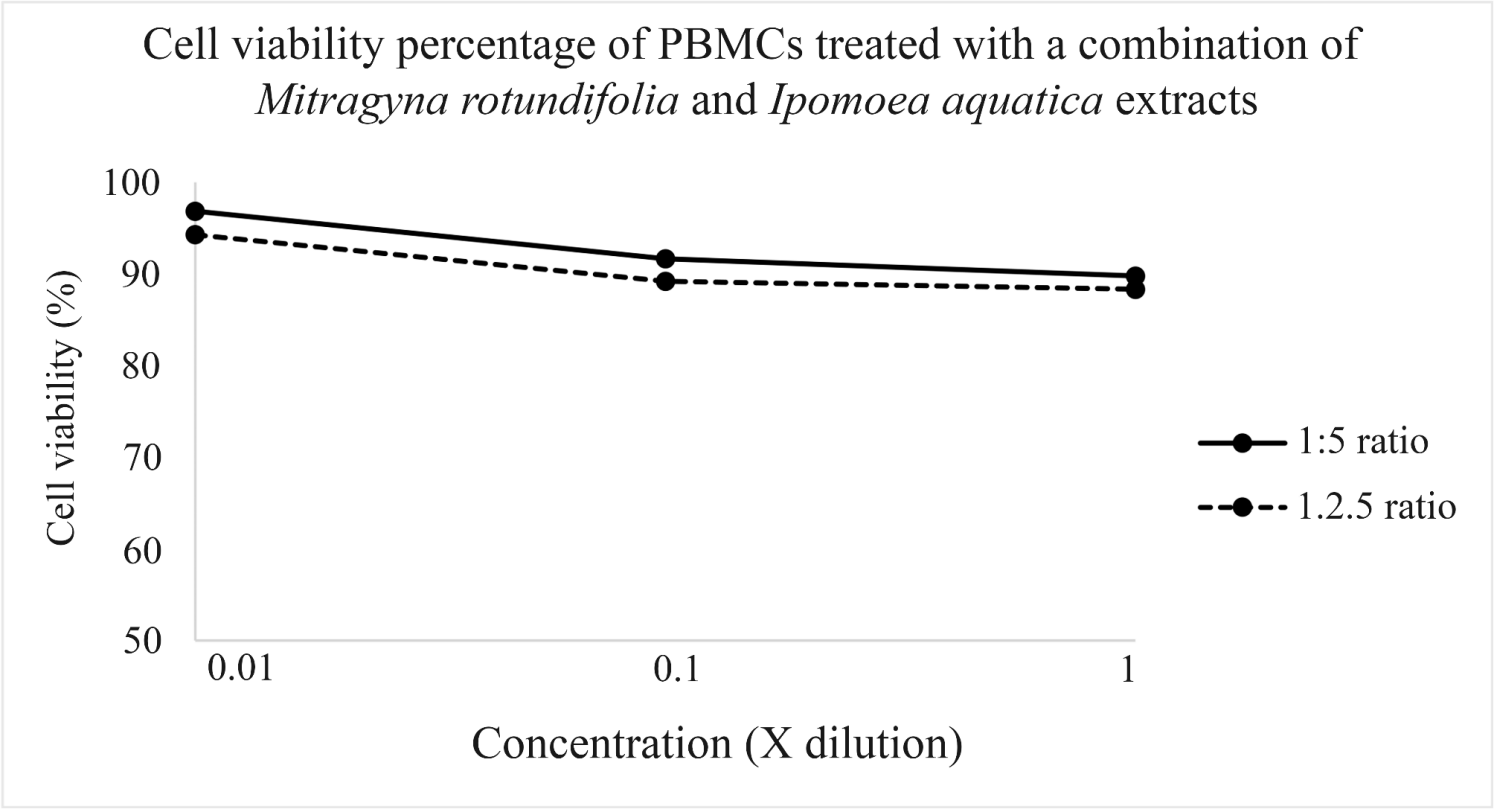
The graph derived from MTT assay, PBMCs treated with the formula of *Mitragyna rotundifolia* and *Ipomoea aquatica* extracts using three working concentrations with a 50/50 mixture, namely, 0.945, 0.0945, 0.00945 mg/ml:1.439, 0.1439, 0.01439 mg/ml (at 1:5 ratio extraction), 1.227, 0.1227, 0.01227: 2.9065, 0.29065, 0.029065 mg/ml (at 1:2.5 ratio extraction), and 1.227 and 2.907 mg/ml showed no toxicity, with no IC_50_ values and high cell viability percentages, 94.28 ± 0.18.

**Fig. 5:**
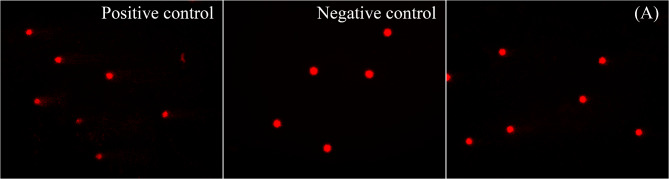
The comet assay images (200x) of PBMC_s_ treated with the highest concentrations used in the MTT assay, 1.227 and 2.907 mg/ml of the *Mitragyna rotundifolia* and *Ipomoea aquatica* mixture extract (A) showing significant DNA damage compared to the negative control.

The formula of *M. rotundifolia* and *I. aquatica* mixed 50/50 with 1:5, and 1:2.5 ratio extractions at 0.945, 0.0945, 0.00945 mg/ml:1.439, 0.1439, 0.01439 mg/ml (for the 1:5 ratio extraction) and 1.227, 0.1227, 0.01227: 2.9065, 0.29065, 0.029065 mg/ml (for the 1:2.5 ratio extraction) were used for testing in the MTT assay. The MTT assay did not have cytotoxicity, indicating the highest cell viability percentage, 94.28 ± 0.18 according to Fig. 4; Table 5. The highest working concentration values used in the MTT assay, 1.227 and 2.907 mg/ml, were used for testing in the comet assay, when no IC_50_ values were found. Significant (*p* < 0.05) DNA damage was found compared to the negative control revealed by a long tail in the olive tail moment testing, according to Fig. 5; Table 5.

When there are conflicting results between the MTT assay without an IC_50_ value and there is significant DNA damage when using the highest working concentration for the comet assay, this value was used to calculate the LD_50_ value instead of the IC_50_ value. The LD_50_ value is a statistical estimate of the mg toxicant per kg bodyweight required to kill 50% of a rat population^28^. The predicted LD_50_ of *M. rotundifolia* and *I. aquatica* of 1,489.53 and 2,503.06 mg/kg body weight categorized them as WHO Class II moderately hazardous (200-2,000 mg body weight, oral) and class III slightly hazardous (Over 2,000 mg/kg body weight, oral). This means that when consuming 200-2,000 dried *M. rotundifolia* mg per kilogram body weight and more than 2000 dried *I. aquatica* mg per kilogram body weight, they will be toxic to the body.

**Fig. 6:**
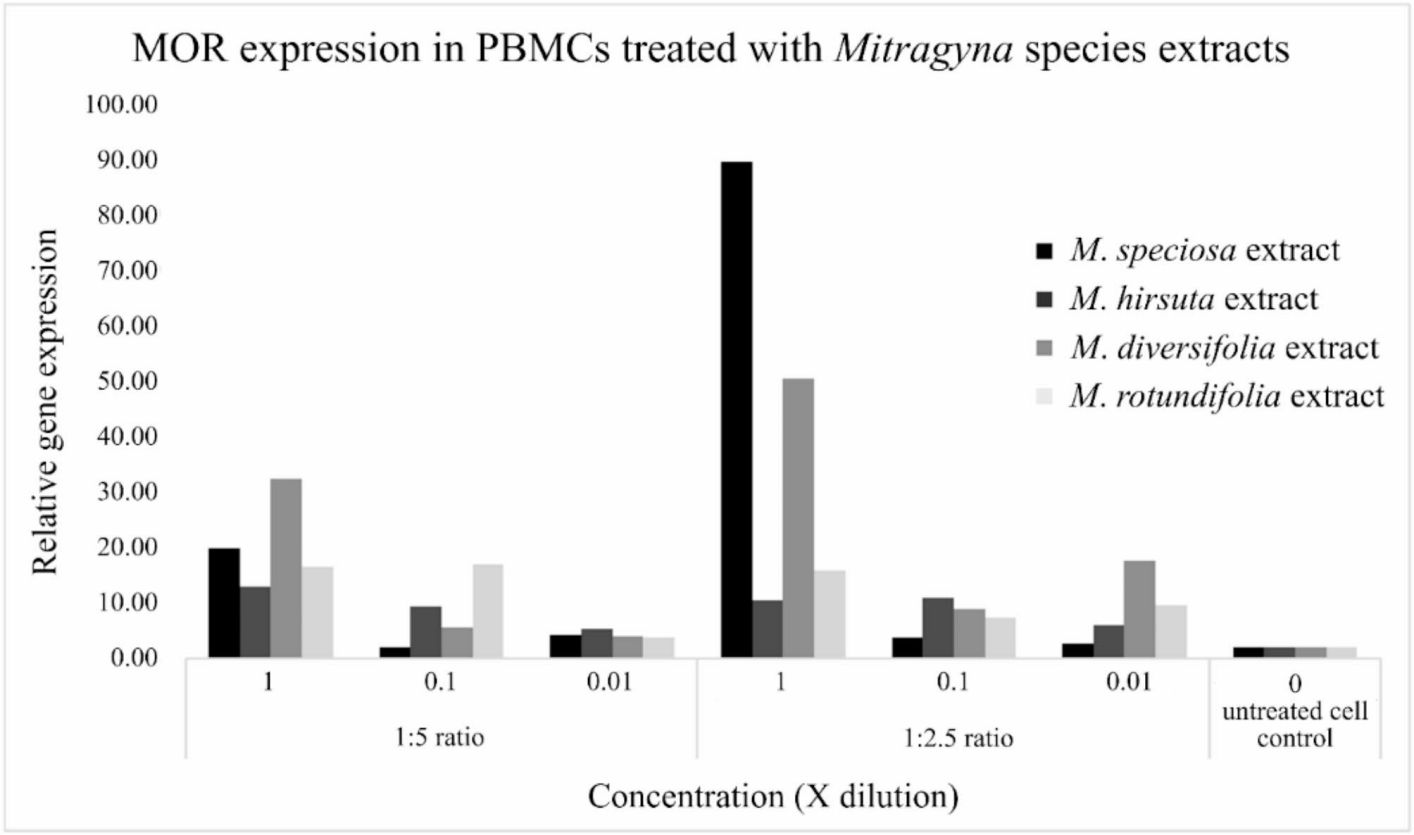
The graph derived from the relative µ (MOR) gene expression of *Mitragyna speciosa*, *M*. *hirsuta*, *M*. *diversifolia* and *M*. *rotundifolia* extracts at various concentrations, the *M*. *speciosa* extract at 1.923 mg/ml showed the highest relative gene expression, 89.54, and the next highest expression, 50.41 for *M. diversifolia* at 1.133 mg/ml (both are in 1:2.5 extraction ratio).

**Fig. 7:**
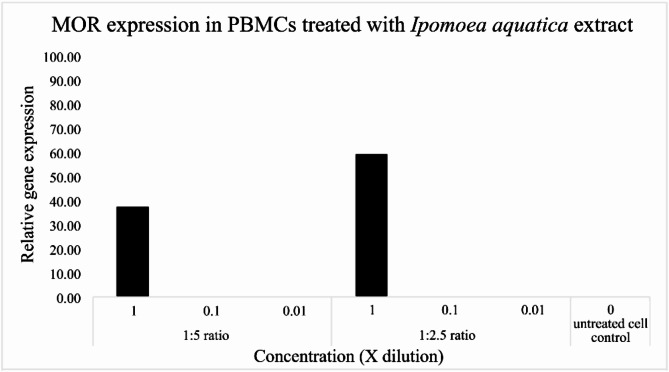
The graph derived from the relative µ (MOR) gene expression of *Ipomoea aquatica* extract at various concentrations, the highest gene expression appeared 37.75–59.76 at concentration of 2.878 and 5.813 mg/ml in 1:5 and 1:2.5 extraction ratios.

**Fig. 8:**
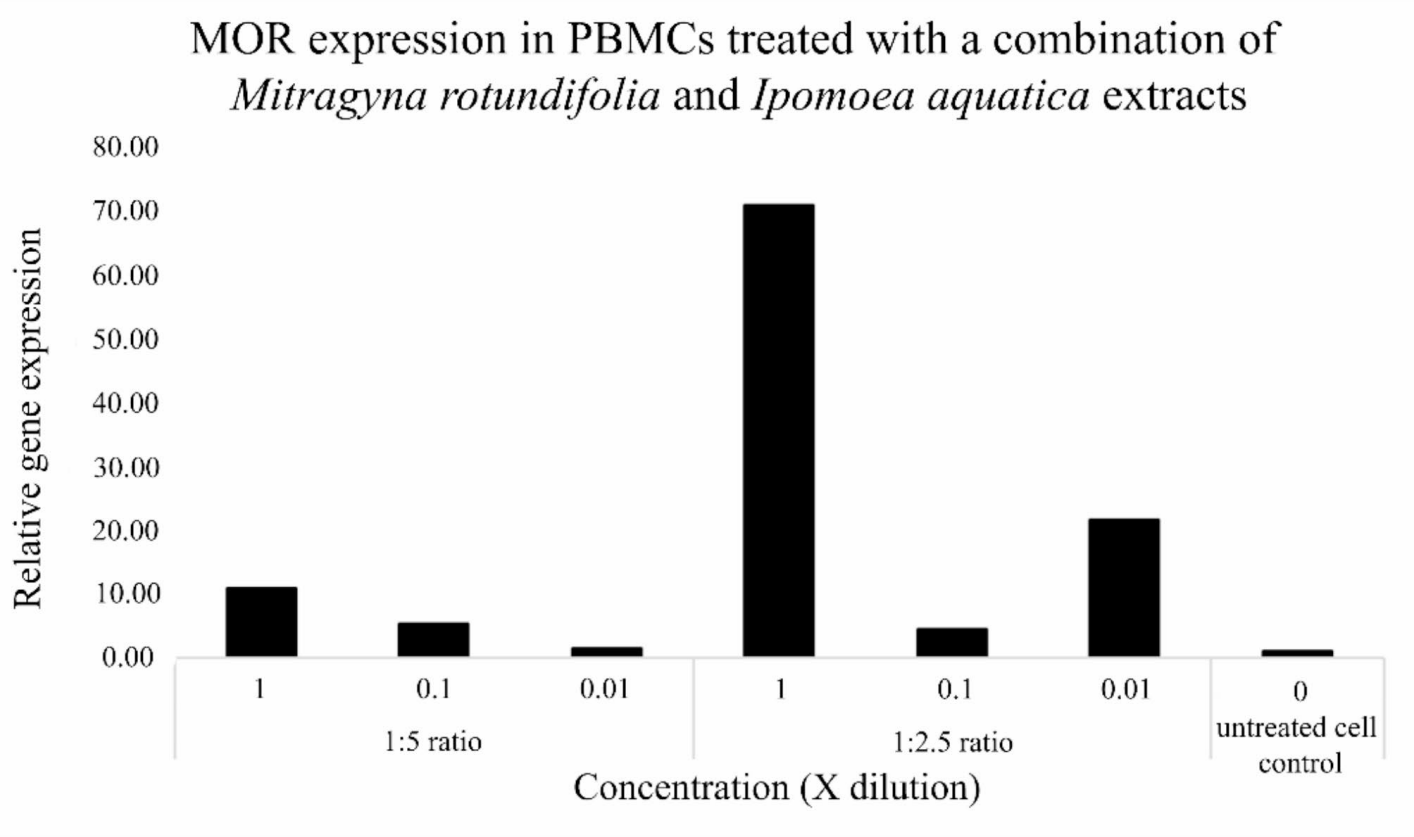
The graph derived from the relative µ (MOR) gene expression of the combination of *M. rotundifolia* and *I. aquatica* extracts showing high expression, 71.01 and 21.71 at concentrations of 1.227, 2.907 mg/ml and 0.0123 and 0.0291 mg/ml (at 1:2.5 mg/ml extraction ratio).

Quantification of human opioid gene expression using the primers µ (MOR), µ (OPRM1), δ (OPRD1), Delta, Kappa, κ (OPRK1) and nociceptin (NOR) on PBMCs treated with the four *Mitragyna* species and *I. aquatica* extracts and the formula indicating that the primer pair µ (MOR) succeeded in DNA amplification with the following conditions: extraction ratios of 1:2.5 and 1:5, three concentrations at 1x, 0.1x and 0.01x mg/ml. The biological activity of the four species’ ethanol extracts showed a binding affinity to the µ (MOR) receptor only. The highest relative gene expression was found at 89.54 and 50.41 by *M. speciosa* and *M. diversifolia* at 1.923 and 1.133 mg/ml, 32.42 and 19.97 by *M. diversifolia* and *M. speciosa* at 3.770 and 1.760 mg/ml. *M. rotundifolia* which contains the three opioids mentioned plus rhynchophylline showed low relative µ (MOR) gene expression of 16.89 at 0.189 mg/ml (Fig. 6), while *I. aquatica* showed higher relative µ (MOR) gene expression than *M. rotundifolia*, 37.75 and 59.76 at 2.878 and 5.813 mg/ml (Fig. 7). After the highest relative µ (MOR) gene expression of the single *M. speciosa* species, the next highest is the formula of *M. rotundifolia* (Mr) and *I. aquatica* (Ia), 71.01 at concentrations of 1.227 (Mr) and 2.907 (Ia) mg/ml and 21.71 at concentrations of 0.0123 (Mr) and 0.0291 (Ia) mg/ml according to Fig. 8. Details of concentrations, relative µ (MOR) gene expression of each *Mitragyna* and *Ipomoea* species are shown in the Table 6. (Ia)

**Table 6:** The relative gene expression of the mu-opioid receptor gene when treated with *Mitragyna* species and *Ipomoea aquatica* extracts, and their combinations, at various extraction ratios and concentrations.

Ratio of extraction	Plant extract	Concentration (mg/ml) (× dilution)	Relative gene expression (2^−ΔΔCP^)
1:5 (g: ml)	*Mitragyna speciosa*	1.76 (1×)	19.97
		0.176 (0.1×)	2.05
		0.0176 (0.01×)	4.17
	*M. hirsuta*	1.560 (1×)	12.98
		0.1560 (0.1×)	9.4
		0.01560 (0.01×)	5.34
	*M. diversifolia*	3.770 (1×)	32.42
		0.3770 (0.1×)	5.63
		0.03770 (0.01×)	3.95
	*M. rotundifolia*	1.890 (1×)	16.6
		0.1890 (0.1×)	16.89
		0.01890 (0.01×)	3.91
	*Ipomoea aquatica*	2.878 (1×)	37.75
		0.2878 (0.1×)	ND
		0.02878 (0.01×)	ND
	*M. rotundifolia* + *I. aquatica*	1×	10.85
		0.1×	5.28
		0.01×	1.39
1:2.5	*M. speciosa*	1.923 (1×)	89.54
		0.1923 (0.1×)	3.75
		0.01923 (0.01×)	2.6
	*M. hirsuta*	0.650 (1×)	10.48
		0.0650 (0.1×)	11
		0.00650 (0.01×)	5.97
	*M. diversifolia*	1.133 (1×)	50.41
		0.1133 (0.1×)	8.92
		0.01133 (0.01×)	17.56
	*M. rotundifolia*	2.454 (1×)	15.79
		0.2454 (0.1×)	7.45
		0.02454 (0.01×)	9.62
	*I. aquatica*	5.813 (1×)	59.76
		0.5813 (0.1×)	ND
		0.05813 (0.01×)	ND
	*M. rotundifolia* + *I. aquatica*	1 × (1.227, 2.907)	71.01
		0.1 × (0.123, 0.291)	4.47
		0.01 × (0.0123, 0.0291)	21.71
Control	Untreated cell	0	1.92

### Discussion and conclusion

There are many previous published papers on various aspects of *M. speciosa* such as its chemical composition and biological effect^3^; *M. speciosa* and mitragynine and its derivatives; its physiological and behavioral effects related to use, abuse, and addiction^2^; biological activity; and toxicity^29^. However, the other *Mitragyna* species have not been covered previously. Key substances that have an impact on human life are mitragynine, 7-hydroxy mitragynine, mitraphylline and rhynchophylline, which are opioid substances. This work is the first to investigate the genus *Mitragyna* to include *M. speciosa*, *M. hirsuta*, *M. rotundifolia* and *M. diversifolia* in Thailand. The results of phytochemical analysis by GC-MS and GC-FID are identical to the previously published papers that found the four mentioned major substances. However, our study is the first time rhynchophylline and mitraphylline have been found in *M. rotundifolia* and *M. diversifolia*. Besides *M. speciosa*, rhynchophylline has also been found in *Uncaria rhyncophylla* which possess numerous protective properties such as being anti-inflammatory, neuroprotective, anti-hypertensive, anti-rhythmic, sedative, and a treatment for Alzheimer’s disease^30–32^. It is accepted that these four substances, which are opioids, bind the four types of opioid receptors, including µ (MOR), δ (DOR), κ, (KOR), and nociception (NOR) receptors, to produce morphine-like effects. In this study, the crude extract of the four studied *Mitragyna* species, rather than purified opioid substances, were investigated to observe plant activity. In order to match plant concentrations and opioid receptors (primers used are shown in Table 2), varied plant and solvent ratios were used for extraction, 1 g: 5 ml and 1 g: 2.5 ml, and various concentrations were produced from these, as shown in the Table 1. When qRT-PCR was performed after first applying the extracts to PBMCs, then RNA extraction and cDNA synthesis, the binding affinity succeeded in µ (MOR), yielding different relative gene expression values shown in the Fig. 6; Table 6. As expected, the highest relative µ (MOR) gene expression occurred in *M. speciosa*, 89.54 (Table 6) at a concentration of 1.923 mg/ml, given the presence of important active ingredients, mitragynine and 7-hydroxymitragynine (Tables 3 and 4) and previously published research^2,3^. A single chemical substance, mitragynine exhibited moderate affinity for the µ and κ receptors, whereas 7-hydroxymitragynine had 14× greater binding affinity than mitragynine (quoted as 40 times by Ahmad et al.^4^) and showed strong affinity for the µ receptor^3^. *M. diversifolia*, *M. hirsuta*, and *M. rotundifolia* extracts showed µ (MOR) gene expression 50.41 at concentrations of 1.133 mg/ml, 12.98 at 1.560 mg/ml and 16.89 at 0.189 mg/ml concentrations, respectively. Please note that *M. rotundifolia* does not contain mitragynine, 7-hydroxymitragynine, and mitraphylline, only rhynchophylline. Rhynchophylline has a vasodilatory effect, treats overactive bladder, augments pentobarbital-induced sleeping behaviors and can be a candidate for treating insomnia^26,33,34^. *I. aquatica* was added in this study to find out whether there are other plants that do not contain these four opioids but are still able to bind to the four opioid receptors. The study found that *I. aquatica* also has binding affinity to µ opioid receptor (MOR), showing a relative gene expression up to 57.96 at a concentration of 5.813 mg/ml. There are not any previously published papers reporting that *I. aquatica* contains opioid substances, despite its properties as a medicinal plant, worldwide vegetable and food, but it has been shown to contain high oleamide, 0.74–1.71 mg/ml and 1.10–7.52 mg/g^27^ which is an endocannabinoid, and to have anti-inflammatory activity^23^. Additionally, oleamide was previously reported to relieve stress, improve memory so help against Alzheimer’s disease, induce deep sleep, and be anti-inflammatory^20–22^. When combination of *M. rotundifolia* and *I aquatica* was tested, it showed higher relative µ (MOR) gene expression up to 71.01, whereas each plant had relative µ (MOR) gene expression as mentioned at concentrations of 1.227 and 2.907 mg/ml, respectively. This means that the combination functions as a µ-opioid receptor (MOR) agonist. The µ-opioid receptor (MOR) agonists, including morphine, remain the most potent analgesics to treat patients with moderate to severe pain. However, the utility of MOR agonists is limited by the adverse effects associated with the use of these drugs, including analgesic tolerance and physical dependence. Therefore, even though the combination showed lower µ-opioid (MOR) gene expression (71.01) than *M. speciosa* (89.54), the combination proved more useful because it does not have the side effects of a drug when used at a rate that would be an overdose of *M. speciosa*. More than the most potent analgesics which treat patients with moderate to severe pain, the combination of the two species *M. rotundifolia* and *I aquatica* which contains both rhynchophylline and oleamide along with the properties of both substances of inducing sleep, increasing sleep duration, and deepening sleep are interesting, and should be further studied in a clinical trial.

As the two-plant combination contains the vital phytochemicals rhynchophylline and oleamide and showed high relative µ-opioid (MOR) gene expression of 71.01 without adverse side effects, it was necessary for the combined mixture to be investigated in terms of toxicity, both cytotoxicity and genotoxicity. The mixture showed no toxicity on PBMCs and does not have an IC_50_ value, and a high cell viability percentage of 94.28 ± 0.18, but induced significant DNA damage. The highest working concentrations have been used for LD_50_ evaluation to predict hazardous categories. This predicted an LD_50_ of 1489.53 mg *M. rotundifolia*/kg body weight and 2,503.06 mg *I aquatica*/kg body weight, categorizing them as WHO Class II moderately hazardous (200-2,000 mg/body weight, oral) and class III slightly hazardous (Over 2,000 mg/kg body weight, oral). This means that when consuming 200-2,000 mg dried *M. rotundifolia* per kilogram body weight and more than 2,000 mg dried *I. aquatica* per kilogram body weight, they will be toxic to the body. For a 50-kg person to meet these classes of hazards, they would have to consume 10,000-100,000 mg of dried *M. rotundifolia* and over 100,000 mg of dried *I. aquatica*. For humans, who are quite different from rats both in body size and genetics, it is very difficult to consume such a high dose corresponding to surpassing these thresholds. So, it can be summarized that there is essentially no toxicity for consumption from DNA results.

In summary, the combination of *M. rotundifolia* and *I aquatica*, either as a combined extract or in any processed form, could be given to people who have problems with insomnia, stress, memory loss, and inflammation. The majority of people with these conditions are elderly people, so the knowledge in this research can therefore be truly used for humanity.

## Data Availability

Data is provided within the manuscript.
